# Cholecystokinin—From Local Gut Hormone to Ubiquitous Messenger

**DOI:** 10.3389/fendo.2017.00047

**Published:** 2017-04-13

**Authors:** Jens F. Rehfeld

**Affiliations:** ^1^Department of Clinical Biochemistry, Rigshospitalet, University of Copenhagen, Copenhagen, Denmark

**Keywords:** cholecystokinin, gastrointestinal hormones, neuropeptides, neuroendocrine tumors, tumor markers

## Abstract

Cholecystokinin (CCK) was discovered in 1928 in jejunal extracts as a gallbladder contraction factor. It was later shown to be member of a peptide family, which are all ligands for the CCK_1_ and CCK_2_ receptors. CCK peptides are known to be synthetized in small intestinal endocrine I-cells and cerebral neurons. But in addition, CCK is expressed in several endocrine glands (pituitary cells, thyroid C-cells, pancreatic islets, the adrenals, and the testes); in peripheral nerves; in cortical and medullary kidney cells; in cardial myocytes; and in cells of the immune system. CCK peptides stimulate pancreatic enzyme secretion and growth, gallbladder contraction, and gut motility, satiety and inhibit acid secretion from the stomach. Moreover, they are major neurotransmitters in the brain and the periphery. CCK peptides also stimulate calcitonin, insulin, and glucagon secretion, and they may act as natriuretic peptides in the kidneys. CCK peptides are derived from proCCK with a C-terminal bioactive YMGWMDFamide sequence, in which the Y-residue is partly *O*-sulfated. The plasma forms are CCK-58, -33, -22, and -8, whereas the small CCK-8 and -5 are potent neurotransmitters. Over the last decades, CCK expression has also been encountered in tumors (neuroendocrine tumors, cerebral astrocytomas, gliomas, acoustic neuromas, and specific pediatric tumors). Recently, a metastastic islet cell tumor was found to cause a specific CCKoma syndrome, suggesting that circulating CCK may be a useful tumor marker.

## Introduction

**Figure 1:**
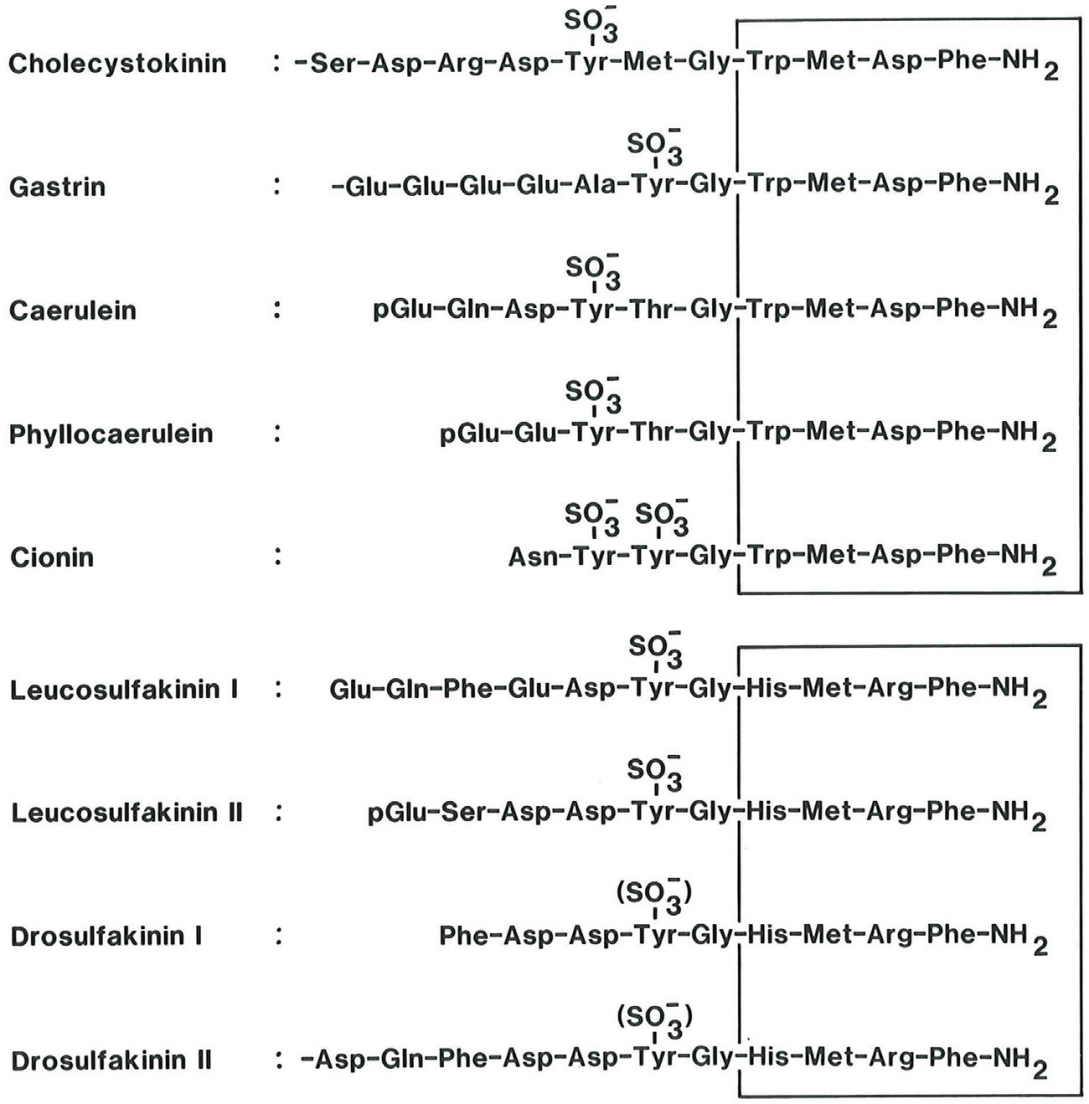
**The homologous bioactive sequences of peptide systems belonging to the cholecystokinin (CCK) family (upper panel)**. CCK and the antral hormone, gastrin, are the only mammalian members of the family. Caerulein and phyllocaerulein are identified from frogskin extracts. Cionin is a neuropeptide isolated from the central ganglion of the protochord, ciona intestinalis. Note the unique disulfated sequence, which might suggest that cionin may resemble a common ancestor of CCK and gastrin. The core of the bioactive sequences, the common C-terminal tetrapeptide amide, is boxed. The lower panel shows the bioactive sequences of the insect peptides, the sulfakinins, which display some homology with vertebrate and protochordian members of the CCK family (4, 5). Also their C-terminal tetrapeptide amide sequence is boxed.

Cholecystokinin (CCK) is member of a family of regulatory peptides with a remarkably well preserved C-terminal sequence (1–3). The family also includes frog skin peptides (caerulein and phyllocaerulein) and the protochordean neuropeptide cionin, but in mammals, CCK and gastrin are the only family members (Figure 1).

After the discovery in 1928 (6), CCK became part of the classical troika of gut hormones together with secretin and gastrin. The last decades, however, have shown that CCK, in addition to its local acute functions in digestion (gallbladder emptying and pancreatic enzyme secretion), is also a growth factor, a neurotransmitter in the brain and peripheral neurons [for reviews, see Ref. (7–9)], and besides, it may be a spermatozoan fertility factor, a natriuretic kidney peptide, an anti-inflammatory cytokine in the immune system, and a cardiac marker of heart failure. The long history has made the CCK literature comprehensive and at some points also confusing because impure CCK preparations with little attention paid to species differences and to physiological levels were used initially. In addition, most assays for measurement of CCK in plasma and elsewhere lacked specificity and sensitivity (10–12).

The biochemical concept of CCK as a single hormonal peptide from the small intestine has also changed considerably. Now CCK is known to be synthetized and released in multiple molecular forms. And the CCK gene is expressed at peptide level in a cell-specific manner in neurons, endocrine cells, and epithelial cells outside the gastrointestinal tract (Table 1). All known biological effects of CCK peptides reside in the conserved C-terminal heptapeptide sequence (Figure 1). Modification of this sequence grossly reduces or abolishes receptor binding and biological effects (13–15). The *N*-terminal extensions of the common C-terminus increase the biological potency and the specificity for receptor binding. Of particular importance is the tyrosyl residue in position seven [as counted from the C-terminus (Figure 1)]. The tyrosyl residue is rarely completely sulfated (16–20). The CCK_2_ receptor binds sulfated and unsulfated ligands equally well, whereas the CCK_1_ receptor is exclusive and requires Y-sulfation of the ligand.

**Table 1:** **The widespread expression of cholecystokinin (CCK) peptides in normal adult mammalian tissue**.

Tissue	Tissue content^a^ (pmol/g)	Precursor percentage^c^
**Intestinal tract**		
Duodenal mucosa	200	5
Jejunal mucosa	150	20
Ileal mucosa	20	50
Colonic mucosa	5	50
**Central nervous system**		
Cerebral cortex	400	2
Hippocampus	350	2
Hypothalamus	200	2
Cerebellum	2	90
Spinal cord	40	10
**Peripheral nervous system**		
Vagal nerve	25	5
Sciatic nerve	15	5
Nerveplexes in colonic wall	5	20
**Extraintestinal endocrine glands**		
Adenohypophysis	25	100
Neurohypophysis	20	10
Thyroid gland	2	20
Adrenal medulla	1	50
**Urogenital tract**		
Renal cortex^b^	+++	−
Renal medulla^b^	+++	−
Testicles	5	80
Spermatozoas	1	50
**Cardiovascular system:**		
Atrial myocytes	10	95
Ventricular myocytes	2	95
**Mononuclear immune cells^b^**	++	−

*^a^Orders of magnitude based on measurement of tissue extracts from different mammalian species*.

*^b^Expression determined only by immunocytochemistry*.

*^c^The precursor percentage was estimated by subtraction of the sum of bioactive, α-amidated CCK peptides (11, 12) from the total procholecystokinin product using the principle of processing-independent analysis (21, 22)*.

The following is a short review about the biology of CCK with emphasis on the recently recognized widespread expression (Table 1) and besides an update on the classic gastrointestinal effects of CCK peptides.

## Biogenesis

As described earlier (9), “*the exomal unit of the CCK gene is seven kilobases interrupted by two introns (**23**). The first of the three exons is small and non-coding. Several conserved regulatory elements have been identified in first 100 bp of the promoter, including an E-box element, a combined cAMP response element (CRE)/12-O-tetradeconoylphorbol-13-acetate response element (TRE), and a GC-rich region (**24*, *25**). Whereas the function of the E-box and the GC-rich region is not fully clarified (**26*, *27**), the combined CRE/TRE sequence plays an important role in the regulation of CCK transcription. The CRE/TRE binds the transcription factor CREB, which is activated by phosphorylation by several signaling pathways, including cAMP, fibroblast growth factor, pituitary adenylate cyclase-activating polypeptide, calcium, hydrolyzates, and peptones to ultimately induce CCK transcription (**28**–**32**). Only one CCK mRNA molecule has been found, and the CCK peptides are thus fragments of the same proCCK protein. The mRNA has 750 bases, of which 345 are protein coding (**33*, *34**). The concentrations of CCK mRNA in cerebrocortical tissue are similar to that of the duodenal mucosa (**34**), and in the brain, there is a rapid synthesis of CCK peptides (**35**)*.

*The primary translational product, preproCCK, has 115 amino acid residues. The first part is the signal peptide. The second part with considerable species variation is a spacer peptide. The bioactive CCK peptides are derived from the subsequent 58 amino acid residues (**16*, *18*, *36**–**38**), and the species variation is small in this sequence. The processing of proCCK is cell-specific: endocrine cells contain a mixture of the medium-sized CCK-58, -33, -22, and -8, whereas neurons mainly release CCK-8 and to some extent CCK-5 (**16*, *39**). The endoproteolysis of proCCK occurs mainly at monobasic sites. Y-77 is mostly O-sulfated (**16**–**20*, *40**), which is decisive for CCK_1_ receptor binding*.

*In the small intestine, CCK peptides are synthesized in endocrine I-cells (**41**), whose apical membrane is in contact with the intestinal lumen and whose basal region contains secretory granules with CCK peptides. CCK is also synthesized in pituitary corticotrophs and melanotrophs, in thyroid C-cells (**17**), and in adrenal medullary cells (**42*, *43**). In the pituitary cells, CCK constitutes a small fraction of the hormones. Tumors originating from pituitary corticotrophs, however, produce larger amounts of CCK (**44**)*.”

It is the brain that expresses most CCK (16, 39, 42). Moreover, cerebral CCK neurons are more abundant than neurons of other neuropeptides (42, 45, 46). While most peptidergic neurons occur in subcortical regions, CCK is expressed in the highest concentrations in neocortical neurons (39, 42, 47). The perikarya of the cortical CCK nerves are distributed in layers II–VI, with the highest frequency in layers II and III (42, 48). CCK in mesencephalic dopamine neurons projecting to the limbic area of the forebrain (45) has aroused clinical interest because these neurons are supposed to be involved in schizophrenia.

Outside the brain, the colon contains numerous CCK neurons, whereas jejunum and ileum are less innervated (42). Colonic CCK fibers occur in the circular muscle layer, which they penetrate to form a plexus in the submucosa (42). In accordance with these locations, CCK peptides excite colonic smooth muscles and release acetylcholine from neurons in both plexus myentericus and submucosa (49). Ganglionic cell somas in pancreatic islets are also surrounded by CCK nerves (50). Moreover, CCK nerve terminals also surround pancreatic islets (51). Finally, afferent vagal nerve fibers also contain CCK (52, 53).

## Endocrine and Neuronal Release

Also mentioned before (9), “*CCK in circulation originates mainly from intestinal endocrine cells. The release to blood was not possible to examine until specific assays were developed (**10**–**12*, *54**). The assays have confirmed that protein- and fat-rich food is the most important stimulus (**11*, *54**). Of the constituents, protein and* *l**-amino acids as well as digested fat cause significant CCK release (**54*, *55**). Carbohydrates only release small amounts of CCK (**54**), but hydrochloric acid also stimulates release (**55**)*.

*The release from neurons has been examined directly in brain slices and synaptosomes (**56*, *57**). Potassium-induced depolarization caused a calcium-dependent release of CCK-8. Similarly, depolarization releases CCK peptides from the hypothalamic dopamine neurons that innervate the intermediate lobe of the pituitary (**58**)*.

*By analogy with other neuropeptides, it is possible that overflow from peripheral CCK neurons may contribute slightly to CCK in plasma.By comparison with identified CCK peptides, it has been possible to deduce the molecular pattern of CCK in plasma. The picture has varied (**12**) due to species differences and because the molecular pattern along the gut varies (**59*, *60**). Furthermore, the distribution may vary during stimulation. In man, CCK-33 predominates in plasma, but CCK-58, -22, and -8 are also present (**11*, *61**)*.

*In the basal state, the concentration of CCK in plasma is around 1 pmol/l, but often less. The concentration increases within 20 min to 3–5 pmol/l during meal stimulation, and then declines gradually only to reach a second peak after 1.5–2 hours. In comparison with most other pancreatic and gastrointestinal hormones (**62**), the concentrations of CCK in plasma are low. When food-induced CCK in plasma is mimicked by infusion of exogenous CCK, the same degree of gallbladder contraction and release of enzymes as seen during meals occurs (**54*, *62**–**64**). Therefore, the low circulating concentrations of CCK are sufficient to account for the gallbladder contraction and pancreatic enzyme secretion during meals*.

*Because the cholecystokinetic and pancreozymic potency of CCK-33 and CCK-8 on a molar base are identical (**65**), it may seem less important what I-cells release during digestion*.” On the other hand, CCK-58, -33, and -22 are cleared from blood at a significantly slower rate than CCK-8.

## Receptors

The cellular effects of CCK peptides are mediated *via* two receptors (66, 67). The “alimentary” CCK-A or CCK_1_ receptor (66) mediates gallbladder contraction, relaxation of the sphincter of Oddi, pancreatic growth and enzyme secretion, delay of gastric emptying, and inhibition of gastric acid secretion *via* fundic somatostatin (68). CCK_1_ receptors have been found also in the anterior pituitary, the myenteric plexus, and areas of the midbrain (69, 70). The CCK_1_ receptor binds with high affinity CCK peptides that are amidated and sulfated, whereas the affinity for non-sulfated CCK peptides and gastrins is negligible.

The CCK-B or CCK_2_ receptor (the “brain” receptor) is the predominant CCK receptor in the brain (67, 71). It is less specific than the CCK_1_ receptor and binds also non-sulfated CCK, gastrins, and C-terminal fragments such as CCK-5. It has been shown that the gastrin receptor cloned from the stomach (67) and CCK_2_ receptors are identical (71, 72). The gastrin/CCK_2_ receptor is expressed also in substantial amounts in pancreatic islet cells in man (73).

## Gastrointestinal Effects

The defining functions of CCKs in digestion have been detailed regularly [for instance, see Ref. (6, 7)].

### *Gallbladder* *and Pancreas*

“*CCK peptides stimulate hepatic secretion mainly as bicarbonate from hepatic ductular cells (**74**) and act on gallbladder muscles with a potency correlated to the low plasma concentrations of sulfated CCK. From the liver and gallbladder, bile is released into the duodenum via CCK-mediated rhythmic contraction and relaxation of muscles in the common bile duct and the sphincter of Oddi. CCK regulates the secretion of pancreatic enzymes so potently that it seems sufficient to account for all enzyme secretion (**63**–**65**). CCK is also capable of releasing several small intestinal enzymes such as alkaline phosphatase (**75**), disaccharidase (**76**), and enterokinase (**77**). In addition, CCK stimulates the biosynthesis of pancreatic amylase, chymotrypsinogen, and trypsinogen (**78**–**80**)*.

*While the interest in the effect of CCK on the exocrine pancreas was for many years restricted to enzyme secretion, it is now well established that CCK also stimulates fluid and bicarbonate secretion. The effect on bicarbonate secretion is in itself weak, but because CCK potentiates the secretin-induced bicarbonate secretion in the same way as secretin potentiates the CCK-induced enzyme release (**81**), the effect of CCK peptides on bicarbonate and fluid secretion is potent. There are species differences, so it is now assumed that CCK in man stimulates pancreatic enzyme secretion through a cholinergic pathway that is less significant in rodents (**82**–**84**)*.

*There are also species differences regarding the endocrine pancreas. CCK peptides release insulin and glucagon more potently in man and pig than in dog and rat (**51*, *85**–**87**). The difference is partly due to neurons in pancreatic islets that release CCK-8 and CCK-5 in man and pig (**51**), whereas rat and dog islets have no such innervation (**50*, *51**). Moreover, islet cells in man and pig also express the CCK_2_ receptor abundantly (**73**), whereas rat islet cells express mainly the CCK_1_ receptor (**88**)*.

*Already in 1967, Rothman and Wells (**80**) noted that CCK increased pancreatic weight and enzyme synthesis. Also the output of bicarbonate and protein from the hypertrophic pancreas was increased (**89**). Although secretin in itself is without trophic effects, the combination of secretin and CCK showed trophic effect on ductular cells with increased secretin-induced bicarbonate output (**89**)*.”

### Gut Motility

Cholecystokinin contributes to control intestinal motility. The distal part of the gut is as mentioned abundantly innervated with CCK neurons (42, 90). It is therefore likely that an increase of intestinal motor activity by exogenous CCK (91) reflects neuronal control of intestinal muscles by CCK peptide transmission. Neuronal CCK acts both indirectly *via* acetylcholine release from postganglionic parasympathetic nerves and directly on muscle cells (49). The observation that CCK peptides stimulate intestinal blood flow is in harmony with the occurrence of CCK nerve terminals around blood vessels in the basal lamina propria and the submucosa (42).

### *Satiety* 

“*In 1973, Gibbs et al. discovered that exogenous CCK inhibits food intake (**92**). The effect mimicked the satiety induced by food and was not seen with other gut peptides known then. The effect could be demonstrated in several mammals. Vagotomy studies indicate that peripheral CCK induces satiety via CCK_1_ receptors relaying the effect into afferent vagal fibers (**93**). The satiety signal then reaches the hypothalamus from the vagus via the nucleus tractus solitarius and area postrema*.

### *Gastric* *Acid Secretion*

*The effect of CCK on gastric acid secretion has been uncertain. On one hand, it has been suggested that intestinal CCK was an acid inhibitor (an enterogastrone). On the other hand, the results of CCK infusions have been inconsistent. The gastrin/CCK double “knockout” mice have now shed further light on the problem showing that circulating CCK stimulates somatostatin release from fundic D-cells via CCK_1_ receptors, which then inhibits acid secretion from parietal cells (**68**)*.”

## Novel Sites of Expression

The major sites of CCK expression are as mentioned endocrine cells in the gut, the brain, and in peripheral nerves. But the last decades have uncovered additional sites and cell types that also express the CCK gene at peptide level (Table 1). In some of these sites, proCCK is not processed to the known α-amidated peptides. Their functions are therefore still unknown. But since CCK receptors also have such widespread expression (66, 67, 70–73, 94, 95), there is both room and need for delineation of the roles of CCK released from the “new” sites.

### Extraintestinal Endocrine Cells

Pituitary corticotrophs and melanotrophs express significant amounts of proCCK fragments, but the posttranslational processing results in only trace amounts of conventional α-amidated CCK peptides (43, 96). Also, thyroid C-cells produce CCK, but mainly as non-sulfated but amidated CCK-8 (17). Since C-cells are well equipped with CCK_2_ receptors (97), thyroid CCK-8 is probably an autocrine stimulator of growth of normal and not least malignant C-cells. Adrenal medullary cells produce small amounts of CCK, although amidated and with a low degree of sulfation (98). The significance of adrenal CCK is unknown.

### Male Germ Cells

Spermatogenic cells express transiently the CCK gene in most mammals (99, 100). Less than 25% of the amidated CCK is sulfated. Interestingly, the CCK peptides in mature spermatozoes are concentrated in the acrosomal granule, which opens the possibility that CCK may play a role in fertilization due to the acrosomal reaction (100). The acrosomal expression is species-specific, as human spermatozoes in addition to CCK also express its homolog, gastrin (101).

### Kidney Cells

In rodent kidneys (rat, mice, and guinea pigs), CCK has recently been shown by immunohistochemistry to be expressed both in the renal cortex and in the medulla. The cortical expression occurs in distal tubular cells and glomeruli, and the medullar CCK expression is confined to collecting ducts (102, 103). The discovery of renal CCK expression may have been stimulated by earlier findings of significant CCK_1_ and CCK_2_ receptor expression also in human kidney tissue (104, 105). It has led to suggestions of local regulatory functions of natriuresis and inflammation in the kidneys. Remarkably, the expression in diabetic mice and rat kidneys is grossly increased. This increase has been suggested to protect the diabetic kidneys somewhat against inflammatory actions of macrophages (103).

### Immune Cells

Cholecystokinin immunoreactivity has consistently been found to be expressed in human and rat mononuclear cells in blood (106, 107). Moreover, CCK-8 (sulfated as well as non-sulfated) has been reported to exert a wide specter of stimulation and inhibition on lymphocytes, macrophages, and cytokine release, with ensuing anti-inflammatory effects (108–111). The field is complex due to the many players; but the clinical impact of CCK in inflammatory diseases and endotoxin shock may be significant.

### Cardiac Myocytes

Fetal mice express high levels of CCK mRNA in cardiac myocytes (112). Accordingly, adult cardiomyocytes in mice, rats, and pigs contain substantial amounts of proCCK protein (113). The processing, however, of cardiac proCCK is unique, as the result is a long triple-sulfated and *N*-terminally truncated fragment 25–94 with only trace amounts of the conventionally amidated and sulfated CCK peptides (113). The tissue concentration of the long proCCK fragment is higher in atrial than ventricular myocytes. The long proCCK fragment is released to plasma and may find use as a marker of the risk of mortality in heart failure patients (113).

### Tumor Expression

Cholecystokinin is expressed at highly variable amounts in different neuroendocrine tumors, especially corticotrophic pituitary tumors (44), medullary thyroid carcinomas (17), phaeochromocytomas (98), and pancreatic islet cell tumors of which some may cause a specific CCKoma syndrome (114–117). CCK is also expressed in Ewing’s Sarcomas, where proCCK measurements may be used to monitor the treatment (118). Cerebral gliomas, astrocytomas, and acoustic neuromas also express CCK (119–121). The present knowledge about tumor expression of CCK was recently summarized in a review that also discussed measurements of CCK and proCCK in plasma as tumor markers (122).

## Conclusion

Since the identification of CCK half a century ago as a single peptide with a sequence of 33 amino acid residues (CCK-33), the CCK story has been loaded with major revelations: first, it was shown that the C-terminus of CCK was similar to that of gastrin, and that CCK and gastrin peptides share the same receptor, the CCK_2_ receptor. Then, it was demonstrated that bioactive CCK occurs in multiple molecular forms—from CCK-58 to CCK-5 with and without tyrosyl O-sulfations. At variable intervals, it has since been shown that CCK peptides are expressed all over the body: in central and peripheral neurons, in intestinal and extraintestinal endocrine cells, in germ cells, kidney epithelial cells, cardiac myocytes, and immune cells. Moreover, the proCCK maturation appears to be cell specific, and tumors expressing CCK release correspondingly varying multifaceted patterns of CCK peptides. Thus, today CCK should be seen as an almost ubiquitous system of intercellular messenger peptides. The complex biology is probably characteristic for many regulatory peptides, for which the CCK system may serve as a source of inspiration for further research.

## Author Contributions

The author confirms being the sole contributor of this work and approved it for publication.

## Conflict of Interest Statement

The author declares that the research was conducted in the absence of any commercial or financial relationships that could be construed as a potential conflict of interest.
